# Brand Name and Generic Proton Pump Inhibitor Prescriptions in the United States: Insights from the National Ambulatory Medical Care Survey (2006–2010)

**DOI:** 10.1155/2015/689531

**Published:** 2015-02-05

**Authors:** Andrew J. Gawron, Joseph Feinglass, John E. Pandolfino, Bruce K. Tan, Michiel J. Bove, Stephanie Shintani-Smith

**Affiliations:** ^1^Division of Gastroenterology & Hepatology, University of Utah, Salt Lake City, UT 84132, USA; ^2^Center for Healthcare Studies, Feinberg School of Medicine, Northwestern University, Chicago, IL 60611, USA; ^3^Division of General Internal Medicine and Geriatrics, Feinberg School of Medicine, Northwestern University, Chicago, IL 60611, USA; ^4^Division of Gastroenterology & Hepatology, Feinberg School of Medicine, Northwestern University, Chicago, IL 60611, USA; ^5^Department of Otolaryngology, Feinberg School of Medicine, Northwestern University, Chicago, IL 60611, USA

## Abstract

*Introduction*. Proton pump inhibitors (PPI) are one of the most commonly prescribed medication classes with similar efficacy between brand name and generic PPI formulations. *Aims*. We determined demographic, clinical, and practice characteristics associated with brand name PPI prescriptions at ambulatory care visits in the United States. *Methods*. Observational cross sectional analysis using the National Ambulatory Medical Care Survey (NAMCS) of all adult (≥18 yrs of age) ambulatory care visits from 2006 to 2010. PPI prescriptions were identified by using the drug entry code as brand name only or generic available formulations. Descriptive statistics were reported in terms of unweighted patient visits and proportions of encounters with brand name PPI prescriptions. Global chi-square tests were used to compare visits with brand name PPI prescriptions versus generic PPI prescriptions for each measure. Poisson regression was used to determine the incidence rate ratio (IRR) for generic versus brand PPI prescribing. *Results*. A PPI was prescribed at 269.7 million adult ambulatory visits, based on 9,677 unweighted visits, of which 53% were brand name only prescriptions. In 2006, 76.0% of all PPI prescriptions had a brand name only formulation compared to 31.6% of PPI prescriptions in 2010. Visits by patients aged 25–44 years had the greatest proportion of brand name PPI formulations (57.9%). Academic medical centers and physician-owned practices had the greatest proportion of visits with brand name PPI prescriptions (58.9% and 55.6% of visits with a PPI prescription, resp.). There were no significant differences in terms of median income, patient insurance type, or metropolitan status when comparing the proportion of visits with brand name versus generic PPI prescriptions. Poisson regression results showed that practice ownership type was most strongly associated with the likelihood of receiving a brand name PPI over the entire study period. Compared to HMO visits, patient visits at academic medical centers (IRR 4.2, 95% CI 2.2–8.0), physician-owned practices (IRR 3.9, 95% CI 2.1–7.1), and community health centers (IRR 3.6, 95% CI 1.9–6.6) were all more likely to have brand name PPIs. *Conclusion*. PPI prescriptions with brand name only formulations are most strongly associated with physician practice type.

## 1. Introduction

Proton pump inhibitors (PPIs) are the most widely prescribed class of medications in the United States, and they account for >$10 billion in annual health care costs [1]. PPIs are prescribed by a wide range of primary and specialty care clinicians for a range of symptoms associated with acid reflux disease.

The widespread use of PPIs has recently garnered attention from the American Board of Internal Medicine's “Choosing Wisely” campaign to promote appropriate discontinuation of PPIs when appropriate [2]. Multiple guidelines promote efforts to utilize the minimum effective dose and pursue alternative diagnoses or treatments in patients whose symptoms do not respond to PPI therapy [3, 4].

Professional guidelines and “Choosing Wisely” goals are laudable; however, they do not address potential cost-saving measures such as the use of generic rather than brand name PPI formulations. Much of the effort to reduce drug costs in this country has been through direct generic substitution, whereby a brand name drug is replaced with its less costly generic equivalent. The estimated national savings from generic substitution of all outpatient drugs are $6 billion for adults younger than 65 years and $3 billion for older adults [5].

PPI prescribing guidelines do not promote one PPI formulation over others, as evidence shows similar symptom relief between different PPI formulations [6]. The active ingredients in generic drugs are the same as in brand name drugs, and the FDA requires that generic drug manufacturers must prove bioequivalence of a generic and brand name formulation [7]. Cost effectiveness studies have found that generic PPIs economically dominate treatment strategies with more expensive PPI formulations [8].

In light of the potential cost differences yet similar efficacy between generic and brand name PPIs, we sought to determine prescribing patterns for brand name versus generic PPIs at ambulatory care visits in the United States. We analyzed physician practice characteristics associated with prescribing brand name PPIs at ambulatory care visits in the United States, controlled for patients' demographic and clinical characteristics. Nationally representative data about prescribing patterns were used to evaluate change in generic substitution between 2006 and 2010, as well as factors associated with brand name prescription across the entire five-year period.

## 2. Methods

### 2.1. Study Design

This study was granted exempt status by the Northwestern University Institutional Review Board. The study used the National Ambulatory Medical Care Survey (NAMCS), a national survey designed to collect information about the use of ambulatory medical services in the United States [9]. The survey is conducted annually by the US National Center for Health Statistics (NCHS), with the US Bureau of Census as the field data collection agent. It utilizes a multistage probability design that involves sampling areas, physician practices within those areas, and patient visits within practices. This allows for estimation of health services representative of US outpatient visits.

### 2.2. Inclusion Criteria

All adult (≥18 yrs of age) outpatient ambulatory care visits from 2006 to 2010 were included in the study population.

### 2.3. PPI Prescriptions

PPI prescriptions were identified by using the drug entry code developed by the National Center for Health Statistics. PPIs with brand name only formulations available for the entire study period (2006–2010) included esomeprazole (Nexium), rabeprazole (Aciphex), omeprazole/sodium bicarbonate (Zegerid), and dexlansoprazole (Dexilant). Pantoprazole (Protonix) was included as a brand name formulation from 2006 to 2007; a generic formulation became available in late 2007. Lansoprazole (Prevacid) was included as a brand name formulation from 2006 to 2009; a generic formulation became available in 2010. Generic formulations included omeprazole (Prilosec) (2006–2010), lansoprazole (Prevacid) (2010), and pantoprazole (Protonix) (2008–2010).

### 2.4. Measures

Patient measures included year of visit (2006–2010), age category (18–24, 25–44, 45–64, and ≥65 years), sex, race and ethnicity (white non-Hispanic, black non-Hispanic, Hispanic, Latino, or other), census estimates of median zip code income, patient insurance (private, Medicare, Medicaid, other, or unknown), and total number of chronic conditions (0, 1-2, and ≥3). NAMCS imputed values for race and ethnicity were used when data were missing. Physician measures included provider specialty type (primary care, medical specialty, or surgical specialty), region (Northeast, Midwest, South, and West), practice ownership (physician-owned, health maintenance organization, community health center, academic medical center, and other), and practice metropolitan status (metropolitan status or nonmetropolitan status, as defined by the US Office of Management and Budget).

### 2.5. Statistical Analysis and Outcomes

Analyses were performed using Stata SE version 12.1 (College Station, TX) survey commands that account for the complex survey design and sample weights. We present both unweighted and nationally weighted estimates of numbers of visits that included either a brand or generic PPI prescription. Chi-square tests were used to compare the proportion of visits with brand name PPI prescriptions versus generic PPI prescriptions for each patient or physician measure. Physician and patient characteristics associated with generic versus brand PPI prescriptions were retained for the multivariable model of PPI prescribing if univariate significance was *P* < 0.1.

To better approximate relative risk [10], Poisson regression was used to determine the incidence rate ratio (IRR) for generic versus brand PPI prescribing [11]. Estimates of the effects of physician practice characteristics are modeled while simultaneously controlling for year of visit and physician and patient characteristics potentially associated with brand versus generic PPI prescriptions.

## 3. Results

**Figure 1:**
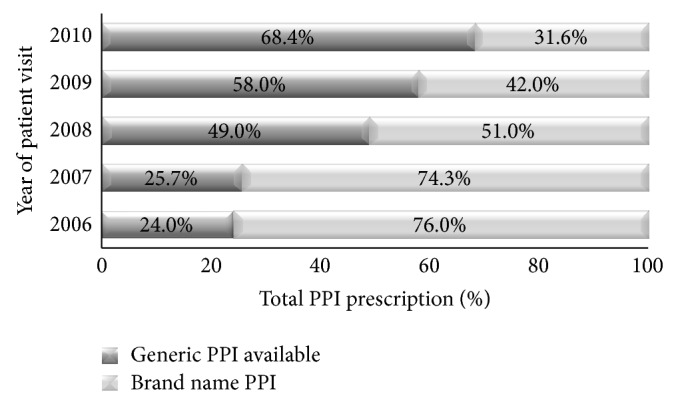
Proton pump inhibitor prescriptions by year of patient visit, NAMCS 2006–2010.

Based on an unweighted total of 9677 NAMCS visits in 2006–2010, a PPI was prescribed at an estimated 329.2 million outpatient adult ambulatory visits nationally from 2006 to 2010. Of these, 53% were brand name prescriptions. The total number of visits with a PPI prescription ranged from a low of 56.7 million visits in 2006 to a high of 79.4 million visits in 2009. As shown in Figure 1, the proportion of brand name only prescriptions decreased over time (*P* ≤ 0.0001). In 2006, 76.0% of all PPI prescriptions had a brand name only formulation; in 2010, 31.6% of all PPI prescriptions had a brand name only formulation available.

Table 1 shows the demographic, socioeconomic, and clinical characteristics of all patient visits and the weighted proportion of visits with brand name compared to generic PPI formulations. In addition to year of visit, there were statistically significant differences in brand name only versus generic formulations associated with patient age category, sex, race and ethnicity, region of the country, and number of chronic conditions (all *P* values < 0.05). There were also statistically significant differences in brand name versus generic formulations associated with provider type and practice ownership (all *P* values < 0.05).

Visits by patients aged 25–44 years had the greatest proportion of brand name PPI formulations (57.9%). There were a greater proportion of female patients who received brand name versus generic PPI prescriptions (55.1% versus 50.9%, *P* = 0.004). There were also a slightly greater proportion of patients with 0 chronic conditions that received brand name PPIs than those with 1-2 or ≥3 chronic conditions (56.5% versus 55.5% versus 52.6%, resp.). Academic medical centers and physician-owned practices had the greatest proportion of visits with brand name PPI prescriptions (58.9% and 55.6% of visits with a PPI prescription, resp.). There were no significant differences in terms of median income, patient insurance type, or metropolitan status when comparing the proportion of visits with brand name versus generic PPI prescriptions (all *P* values > 0.5).

Poisson regression results showed that practice ownership type was strongly associated with the likelihood of receiving a brand name PPI over the entire study period. Compared to HMO visits, patient visits at academic medical centers (IRR 4.2, 95% CI 2.2–8.0), physician-owned practices (IRR 3.9, 95% CI 2.1–7.1), and community health centers (IRR 3.6, 95% CI 1.9–6.6) were all more likely to have brand name PPIs. As reflected in Table 1 and controlled for in the final adjusted model, year of visit was also associated with brand name only formulations. For example, patient visits in 2010 were 60% less likely (IRR 0.4) to have a brand name only prescription compared to visits in 2006. In the final adjusted model (Table 1), patient sex (female), race (black), region of the country (South), provider specialty type (medical and surgical subspecialists), and the total number of chronic conditions (0 versus 1-2 or ≥3) were slightly associated with higher proportion of brand name prescriptions.

## 4. Discussion

Our study demonstrates three principal findings. First, PPI formulations available as only brand name prescriptions accounted for a substantial proportion (>50%) of all PPI prescriptions from 2006 to 2010. It is worth noting that the NAMCS dataset accounts for both prescription and over the counter PPI use [12]. Second, the proportion of brand name only PPI prescriptions has decreased over time, likely due to increased availability of other generic formulations. Third, physician practice ownership is the factor most strongly associated with a brand name PPI prescription in the United States.

A likely reason for the overall decrease in brand name prescriptions over time is simply due to increased number of available generic formulations during the study time period. Omeprazole was the first generic PPI available in 2002. Teva Pharmaceutical Industries LTD launched generic pantoprazole in late 2007. This was done “at risk” as the company was sued for patent infringement by Pfizer but continued to sell its generic version through 2010. Teva eventually agreed to pay $1.6 billon to resolve claims related to the generic launch [13]. A generic formulation of lansoprazole became available in late 2009. Most recently, the US Food and Drug Administration approved a generic version of rabeprazole, which should only continue the trend seen in Figure 1.

There is little data to support efficacy differences between PPI formulations and there are cost differences between brand name and generic formulations of the same class of drugs. Interestingly, one company (AstraZeneca©) manufactures both the most widely prescribed generic PPI formulation (omeprazole) and the most widely prescribed brand name PPI formulation (esomeprazole). Esomeprazole accounted for the largest amount of sales ($5.2 billion dollars in 2010) of not only all PPIs, but also all drugs in the US in 2010 [9]. According to Medi-Span drug data, a 3-month supply of omeprazole 20 mg capsules (delayed release) is estimated to cost $390; esomeprazole 20 mg (delayed release) is estimated to cost $804 [14]. Online generic formulations of omeprazole 20 mg (delayed release) are available in the $13–16 range for 28 tablets [15, 16]. In previous work evaluating the cost effectiveness of seven different PPIs and a variety of treatment regimens, generic omeprazole (20–40 mg daily) was the least costly and most effective at treating gastroesophageal reflux disease and hence dominated all strategies [17]. Generic omeprazole was available during our entire study period, yet, only in 2009 and 2010 did PPIs with an available generic formulation account for a greater proportion of visits than brand name only PPIs.

Much of the effort to reduce national drug costs has been through direct generic substitution, whereby a brand name drug is replaced with its less costly generic equivalent, when available. Loss of patent protection for several brand name drugs and the increasing use of tiered pricing strategies that encourage patients to select lower-cost generic drugs have led this approach to be relatively successful [18, 19]. Duru et al. found that generic substitutions resulted in an average annual saving of $160 in the case of Medicare Low Income Subsidy (LIS) beneficiaries and $127 in the case of non-LIS beneficiaries, suggesting that great opportunities exist to reduce costs to individuals and the government with increased generic substitution. Fischer and Avorn identified potential savings of $229 million that could have been realized from wider generic substitution for Medicaid drug payments from 48 states in 2000 [20].

We found that, from 2006 to 2010, the generic share of total PPI prescriptions increased from 24.0 percent to 68.4 percent in 2010. The increasing generic share of PPIs in our study is in line with studies of all outpatient drugs. Aitken et al. found the generic share of total prescriptions increased from 51 percent in 2002 to 67 percent in 2007 [19]. However, 31.6% of PPIs prescriptions were for brand name formulations in 2010, suggesting that there is still opportunity for increased direct generic substitution for PPIs. Variations in care point to opportunities to improve quality.

Our study identified practice ownership as the greatest predictor of brand name PPI prescribing. A prior study of 350,000 physicians in Sweden found that physicians working at private practices were 50–80% more likely to oppose generic substitution than county-employed physicians working on salary [21]. The researchers also found that the probability of a veto of generic substitution increased as patients' copayments decreased [21]. We also found that academic medical practices were also associated with higher rates of brand name PPI prescribing. Physicians and trainees in the US are often unaware of the costs of medical care, and numerous factors make acquiring cost information difficult. Okike et al. found that attending surgeons correctly estimated the cost of commonly used orthopedic devices only 21% of the time [22]. Studies by Epstein et al. [23] and King et al. [24] showed that restricting access of marketing representatives to trainees reduced subsequent prescribing of high-cost but low-value brand name psychoactive drugs. A national survey of United States physician trainees showed an association between positive attitudes toward industry-physician interactions, less knowledge about evidence-based prescribing, and greater inclination to recommend brand name drugs [25]. Recent policy changes seek to reduce undue influence of pharmaceutical marketing on physicians. Particular attention has been paid to trainees because the medical school and residency learning environment may influence subsequent professional development and behavior [25]. The impact on these policies and on brand name versus generic prescribing remains to be determined.

A variety of other factors may also influence generic substitution [5]. Generic substitution is regulated by state laws and many states allow pharmacists to substitute a generic unless explicitly directed by the physician or patient. However, few states mandate that a pharmacist substitute a generic unless overridden by a physician's order [26]. Second, payment structure may encourage or discourage generic substitution by assigning lower or higher out-of-pocket costs to generic formulations. Prior studies show inconsistent results on patients' perspectives on the perceived efficacy or safety of generic versus brand name drugs [27–29].

This was a retrospective analysis using NAMCS data, which uses a robust survey design to help ensure that trends identified are reflective of all outpatient physician visits. Study limitations include the possibility of sampling and misclassification bias. Our data reflect PPIs that have available generic formulations, and it is possible that many prescriptions provided by physicians did not actually result in generic substitution. NAMCS physician visit records only list up to 8 medications per visit. It is possible that patients with greater than eight medications could be taking medications not listed, including PPIs. This would lead to an underestimation of PPI prescriptions. We were unable to control for state or local variables that could provide more granular regional detail of prescribing practices. NAMCS data also do not provide patient level data on severity or chronicity of patient symptoms.

Diagnoses are listed in the NAMCS data; however, discrete diagnoses are not linked to each medication and we were unable to account for the specific diagnoses or symptoms that could be driving PPI prescriptions with only brand name formulations. For example, initial clinical cohort studies in 2009 reported an increased risk for adverse cardiovascular events, when under simultaneous clopidogrel and PPI treatment due to CYP2C19 inhibition. This led the United States Food and Drug Administration and the European Medicines Agency to discourage the combination of clopidogrel and PPI (particularly omeprazole). Subsequent studies including propensity score matching and/or randomization showed contradictory results [30]. Pharmacokinetic and pharmacodynamic data have suggested varying inhibition by different PPIs of the enzyme systems necessary to convert clopidogrel to its active form, but there is no high level evidence that differences on surrogate markers translate into meaningful differences in outcomes [31].

## 5. Conclusion

Although use of PPIs with generic formulation availability is increasing, there still appears to be opportunity for significant improvement. Physicians and other mid-level providers have the “power of the pen” [32] and thus a responsibility to ensure that the least costly medications are available to patients when efficacy is similar for different formulations for the same class of drugs.

## Figures and Tables

**Table 1:** Predictors of brand name proton pump inhibitor choice among patients prescribed a proton pump inhibitor, 2006–2010.

	Total unweighted sample (*N* = 9,677)	Weighted encounters with a brand name PPI, millions (%)^†^	*P* value	Adjusted incidence rate ratio (95% CI) of brand name PPI
Year				
2006	1753	43.1 (76.0)	**<0.0001**	Ref
2007	2011	46.2 (74.3)		1.0 (0.9-1.0)
2008	1665	31.8 (51.0)		0.7 (0.6-0.7)
2009	2153	33.3 (42.0)		0.6 (0.5-0.6)
2010	2095	21.7 (31.6)		0.4 (0.4-0.5)
Age category, y				
18–24 yrs	144	2.2 (46.1)	**0.004**	Ref
25–44 yrs	1383	26.9 (57.9)		1.2 (0.9–1.5)
45–64 yrs	3766	70.0 (55.4)		1.2 (0.9–1.5)
≥65 yrs	4384	76.9 (50.7)		1.1 (0.9–1.4)
Sex				
Female	5857	111.4 (55.1)	**0.004**	Ref
Male	3820	64.6 (50.9)		0.9 (0.9-1.0)
Race/ethnicity				
White	7478	133.3 (51.4)	**0.001**	Ref
Black	862	17.6 (59.8)		1.1 (1.0–1.2)
Hispanic/Latino	972	12.4 (38.8)		1.1 (1.0–1.2)
Other	365	5.6 (51.4)		1.0 (0.8–1.1)
Median Income				
Q1 (<32.8 K)	2091	37.5 (57.0)	0.2	
Q2 (32.8–40.6)	2340	42.7 (53.2)		
Q3 (>40.6–52.4)	2375	42.9 (52.7)		
Q4 (>52.4)	2339	42.6 (51.5)		
Region				
South	3318	80.0 (60.0)	**<0.0001**	Ref
Northeast	1916	34.7 (53.8)		0.9 (0.8–1.0)
Midwest	2515	34.6 (45.8)		0.8 (0.7–0.9)
West	1928	26.8 (47.9)		0.8 (0.8-0.9)
Patient insurance type				
Private	3920	77.3 (54.3)	0.1	
Medicare	4009	71.1 (51.4)		
Medicaid	839	13.7 (58.3)		
Other	615	9.0 (56.8)		
Unknown	294	4.9 (53.2)		
Total number of chronic conditions				
0	1917	36.6 (56.5)	**0.0001**	Ref
1-2	4610	86.9 (55.5)		1.0 (1.0-1.1)
≥3	3150	52.6 (48.8)		0.9 (0.9-1.0)
Provider type				
Primary care	4426	95.0 (50.9)	**0.002**	Ref
Medical spec.	3436	59.0 (57.1)		1.1 (1.0–1.2)
Surgical spec.	1815	22.1 (56.0)		1.1 (1.0-1.1)
Practice ownership				
HMO	217	0.7 (13.8)	**<0.0001**	Ref
Physician-owned	7174	148.9 (55.6)		3.6 (2.0–6.4)
Community health center	982	4.0 (52.5)		3.7 (2.0–6.7)
Academic medical center	197	3.9 (58.9)		4.0 (2.2–7.3)
Other	1093	17.8 (43.4)		3.2 (1.8–5.6)
Metropolitan status of practice				
MSA	8342	151.3 (53.7)	0.6	
Non-MSA	1335	24.7 (52.2)		

^†^As a percentage of the total number of patients prescribed a PPI, adjusted for sample weights.

## Prior Presentation

The results from this paper were presented as an oral presentation on May 6, 2014 at Digestive Disease Week (DDW) in Chicago, IL, USA.
